# The evolution of molecular machines through interfacial nanoarchitectonics: from toys to tools

**DOI:** 10.1039/d0sc03164j

**Published:** 2020-07-08

**Authors:** Katsuhiko Ariga

**Affiliations:** WPI Research Center for Materials Nanoarchitectonics (MANA), National Institute for Materials Science (NIMS) 1-1 Namiki Tsukuba Ibaraki 305-0044 Japan ARIGA.Katsuhiko@nims.go.jp; Graduate School of Frontier Sciences, The University of Tokyo 5-1-5 Kashiwanoha Kashiwa Chiba 277-8561 Japan

## Abstract

Molecular machines are often regarded as molecular artworks and sometimes as fancy molecular toys. However, many researchers strive to operate molecular machines as useful tools for realistic practical applications. In this perspective article, shifting the working environment of molecular machines from solution to interfacial media is discussed from the viewpoint of their evolution from scientific toys to useful tools. Following a short description of traditional research into molecular machines in solution and their nanotechnological manipulation on clean solid surfaces, pioneering research into molecular machine operation at dynamic interfaces, such as liquid surfaces, is discussed, along with cutting-edge research into molecular machine functions in living cells and their models. Biomolecular machines within organisms are the products of evolution over billions of years. We may nanoarchitect such sophisticated functional systems with artificial molecular machines within much shorter periods.

## Abstract 2

Molecular machines can be made more useful with interfacial nanoarchitectonics.

## Graphical abstract

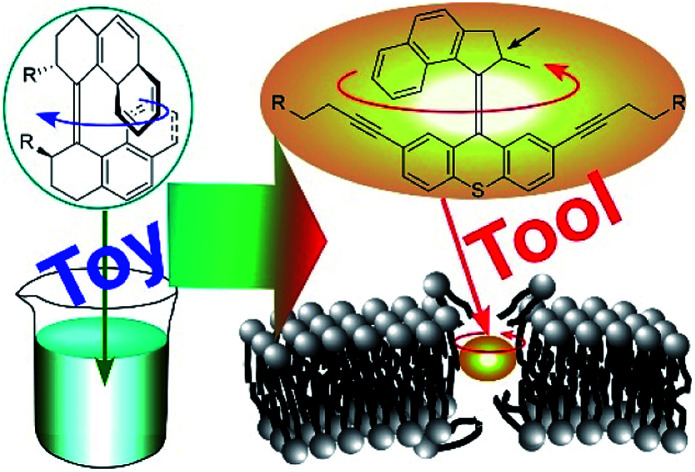

## Introduction

For various issues facing society, including energy,^1–4^ environmental,^5–8^ and biomedical^9–12^ problems, the development of sophisticated material systems with superior efficiency and specificity is required. In addition to improving the intrinsic properties of materials themselves, precise structural control of material components and their organization is an undoubtedly important factor for optimizing their function.^13–16^ Ideal functional systems can be seen in biological systems, in which highly complex functions are commonly found, as exemplified by photosynthetic processes and signal transduction pathways.^17,18^ Functional molecules such as proteins and nucleic acids co-operatively work like machines and devices. Learning from these biological examples, molecular machines and molecular devices could also be key players in achieving highly sophisticated materials to meet our societal demands.

The importance of molecular machines was recognized in previous centuries. With pioneering research efforts for the design and synthesis of molecular machines, the 2016 Nobel Prize in Chemistry was awarded to Jean-Pierre Sauvage,^19^ Sir J. Fraser Stoddart^20^ and Bernard L. Feringa.^21^ In addition to the work of these awardees, excellent examples of molecules with machine-like functions have been continuously reported.^22–31^ Some of these molecules can be regarded as molecular artworks and sometimes as fancy molecular toys. However, many researchers have dreams to operate molecular machines as useful tools in realistic practical applications, although it is not an easy process. High-level control of molecular machines and their sophisticated organization would be required to accomplish this dream.

Molecular machines originated as products of transitional chemistry fields, such as organic chemistry and supramolecular chemistry, where the observation of a single molecular machine is not always easy. This insufficiency is fortunately compensated for by developments in nanotechnology. Various probe microscopies and advanced analytical methods now enable us to directly observe, analyze, and manipulate a single molecular machine.^32,33^

For the next stage, the organization of molecular machines using nanotechnology techniques is necessary. This new phase of molecular machine research can be supported by an emerging concept called nanoarchitectonics,^34–36^ which couples nanotechnology and other research fields including organic chemistry, supramolecular chemistry, and biochemistry, as pioneered by Masakazu Aono.^37,38^ The aim of nanoarchitectonics is to use molecular units to construct functional material systems with advanced features, which can be fabricated into interfacial structures exhibiting hierarchical organization.^39–41^ In order to make molecular machines really useful, they have to be fixed and organized onto defined sites such as interfaces. Interfaces are beneficial environments for connecting molecular functions with device outputs and biological mechanisms.^42–44^ Thus, molecular machines can evolve from toys to tools through the use of interfacial nanoarchitectonics.

**Fig. 1:**
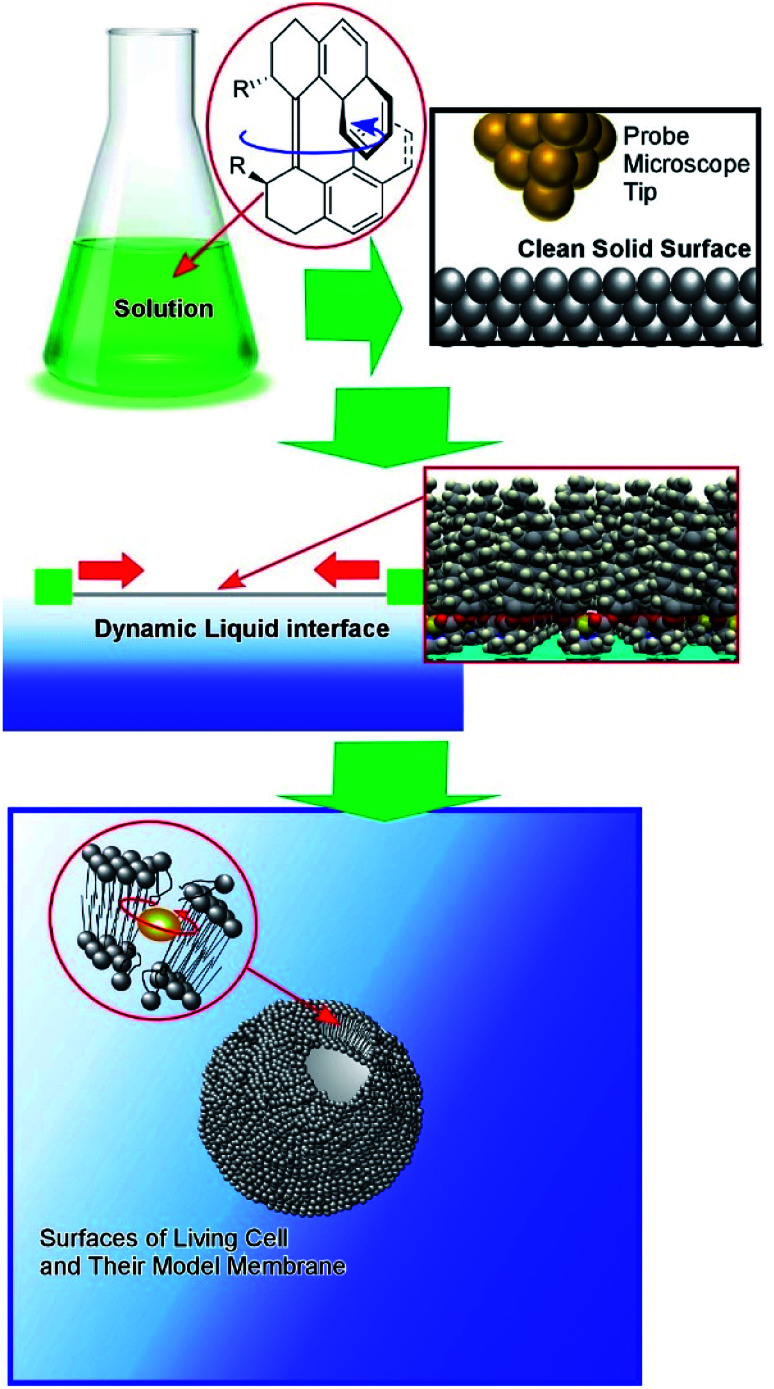
The shift in the working environments of molecular machines, for the evolution of molecular machines from scientific toys to useful tools.

In this perspective article, a shift of the working environments of molecular machines from solution to interfacial media is discussed from the viewpoint of the evolution of molecular machines from scientific toys to useful tools (Fig. 1). Following a short description of traditional research into molecular machines in solution and their nanotechnological manipulation at clean solid surfaces, pioneering research into molecular machine operation at dynamic interfaces such as liquid surfaces is introduced, in addition to cutting-edge research into molecular machine functions in living cells and their models.

## Molecular machines in solution

**Fig. 2:**
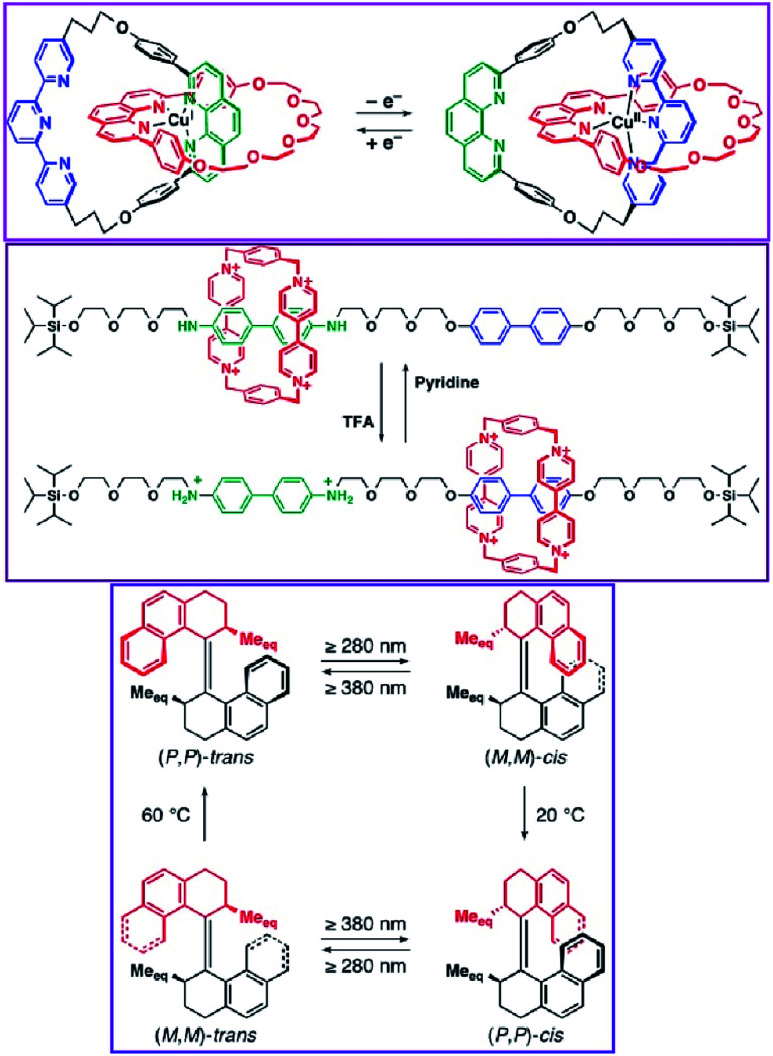
Pioneering examples of molecular machines (a catenane, a molecular shuttle, and a molecular motor). These schemes were provided courtesy of Dr Masayuki Takeuchi, National institute for Materials Science.

Pioneering operations of molecular machines were demonstrated in solution, where intentional motions of well-designed molecules and supermolecules were induced upon application of external stimuli. As exemplified by the Nobel prize machines (Fig. 2), rotational re-arrangement of integrated molecular rings (catenane),^45,46^ switching of ring positions at different stations within a rotaxane chain (molecular shuttle),^47^ and stepwise and directional rotation of half of a molecule relative to the remaining half (molecular rotor and/or molecular motor),^48^ were realized with inputs of external stimuli. These examples can be regarded as masterpiece molecules, achieved through expert design employing organic chemistry and stimulus-responsive strategies in supramolecular chemistry.

**Fig. 3:**
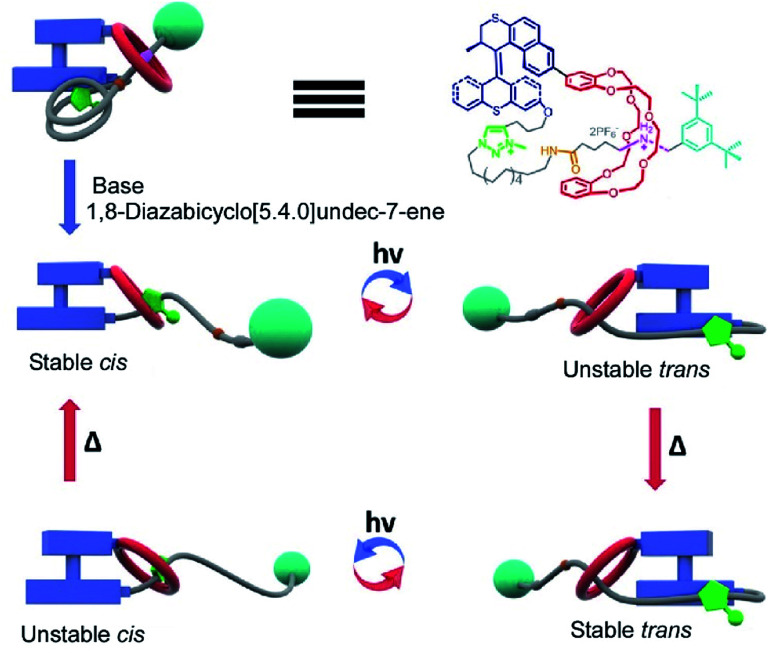
A molecular machine with the coupling of the translational motion of a molecular shuttle and the rotational motion of a molecular motor. Reproduced with permission ref. 49. Copyright 2019, American Chemical Society.

**Fig. 4:**
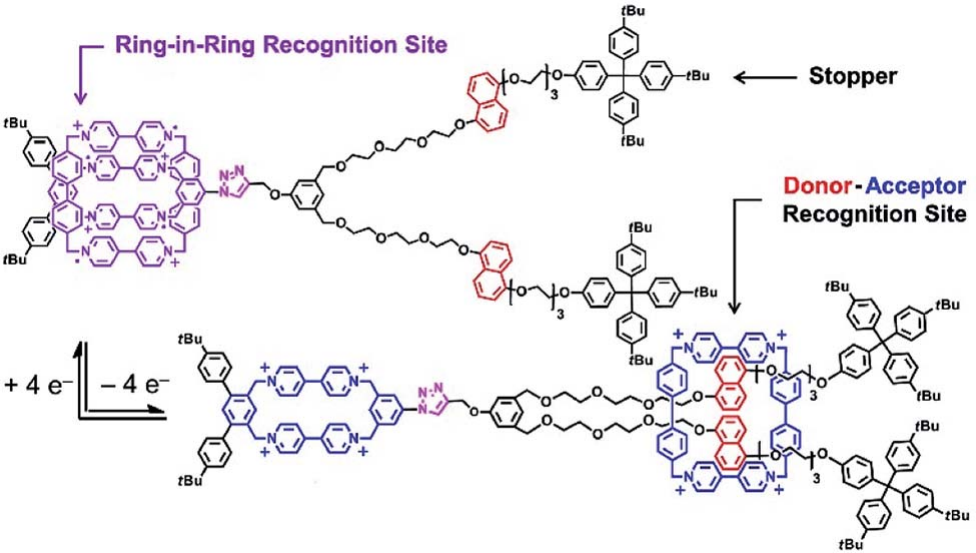
A molecular shuttle with branched chain structures for shape shifts, named a molecular zipper. Reproduced with permission ref. 50. Copyright 2019, American Chemical Society.

Molecular machines in solution environments have been actively researched on the strong basis of organic chemistry and supramolecular chemistry. Undoubtedly, these research activities support the fundamental development of molecular machine science. Various designs and motion patterns of molecular machines have been proposed. For example, Qu and co-workers successfully synthesized a molecular machine, which exhibits a coupling of the translational motion of a molecular shuttle and rotational motion of a molecular motor (Fig. 3).^49^ A ring part of a bistable [1]rotaxane unit is also linked to a molecular motor. Photo-driven *cis*–*trans* isomerization of the motor induces a sliding motion of the molecular shuttle. The use of nanoarchitectonics to couple different molecular motions is an important step in combining individual machines into a unified functional system. Well-considered molecular design sometimes induces larger amplitude molecular motions by the same operational principal. The molecular shuttle shown in Fig. 4 possesses branched chain structures, which enable shuttle motions between two stations to cause larger molecular shape shifts named molecular zippers.^50^ This type of wisely designed nanoarchitectonics would be useful to convert molecular motion into larger actions such as material actuation.

## Molecular machines on clean solid surfaces

As another playground for molecular machines, clean solid surfaces are recognized as ideal environments for the evaluation of molecular shapes and motions with excellent structural precision. Although in most cases operational conditions are impractical, such as ultralow temperature and high vacuum, the direct and precise observation of a single molecule provides indispensable insights for molecular machine science.^51,52^ Experimental approaches under ultra-high vacuum can avoid the contribution of Brownian motion. High-resolution observations of molecular machines are usually obtained with scanning tunneling microscopy (STM). Driving fuels can be electronically supplied from the STM tip.

One representative category of molecular machine research on clean solid surfaces is centered around the nanocar (or molecular car).^53,54^ Nanocars can be regarded as molecular machines with automobile-like motions. Mechanisms of a single molecule were semi-classically explained on the basis of quantum incoherence of their mechanical degrees of freedom by Joachim and Gimzewski.^55,56^ Tour and co-workers demonstrated that the motional mechanisms of their nanocar on a solid surface are almost classical under specific conditions.^57^ Built upon these basic understandings, molecular machine operations on clean solid surfaces have progressed in the last decade. As a demonstration of progress in this research field, the worldwide nanocar race was held in 2017, with 6 teams from France, Germany, Switzerland, USA, USA/Austria, and Japan.^58,59^ Operations of nanocars with regulated translational motions driven by electrical stimuli from the STM tip without touching the nanocar molecule were demonstrated in public.

**Fig. 5:**
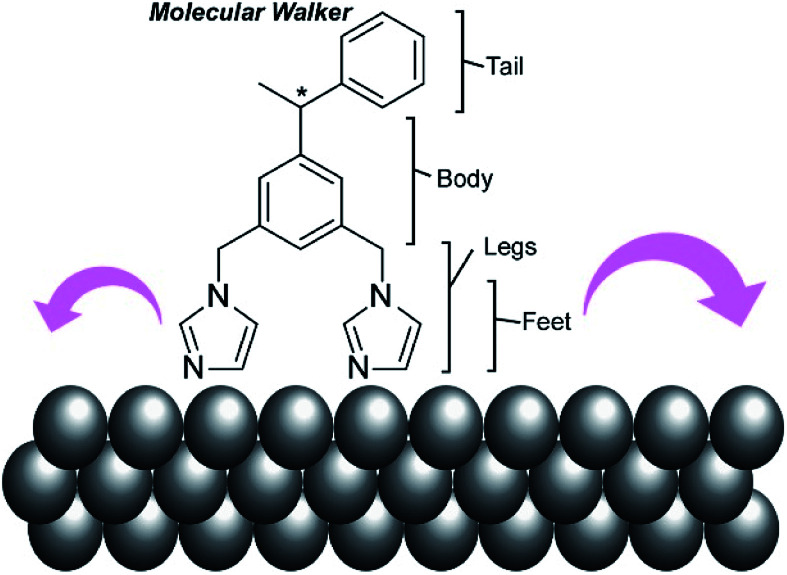
A chiral molecular walker, 1,3-bis(imidazol-1-ylmethyl)-5(1-phenylethyl)benzene, on a Cu(110) surface.

In addition to experimental work, simulation approaches have been applied to figure out the principles of molecular machine motions, because a single molecule fixed on a clean solid surface is an ideal situation for theoretical research. For example, Abbasi-Pérez, Kantorovich, and co-workers simulated the motions of chiral molecular walkers on a clean solid surface.^60^ The diffusive motions of the chiral molecular walker, 1,3-bis(imidazol-1-ylmethyl)-5(1-phenylethyl)benzene (Fig. 5), on a Cu(110) surface along the Cu rows were estimated by kinetic Monte Carlo simulations. The molecular walker moves from one position to the nearest lattice in an inchworm motion, whereby the walker steps first with the rear foot and next with the front foot. This two-step mechanism with its asymmetric design makes the walker diffuse preferentially in a particular direction with Brownian ratchets. The obtained knowledge is also useful for the separation of enantiomers through controlled diffusion on the surface upon applying appropriate external signals.

**Fig. 6:**
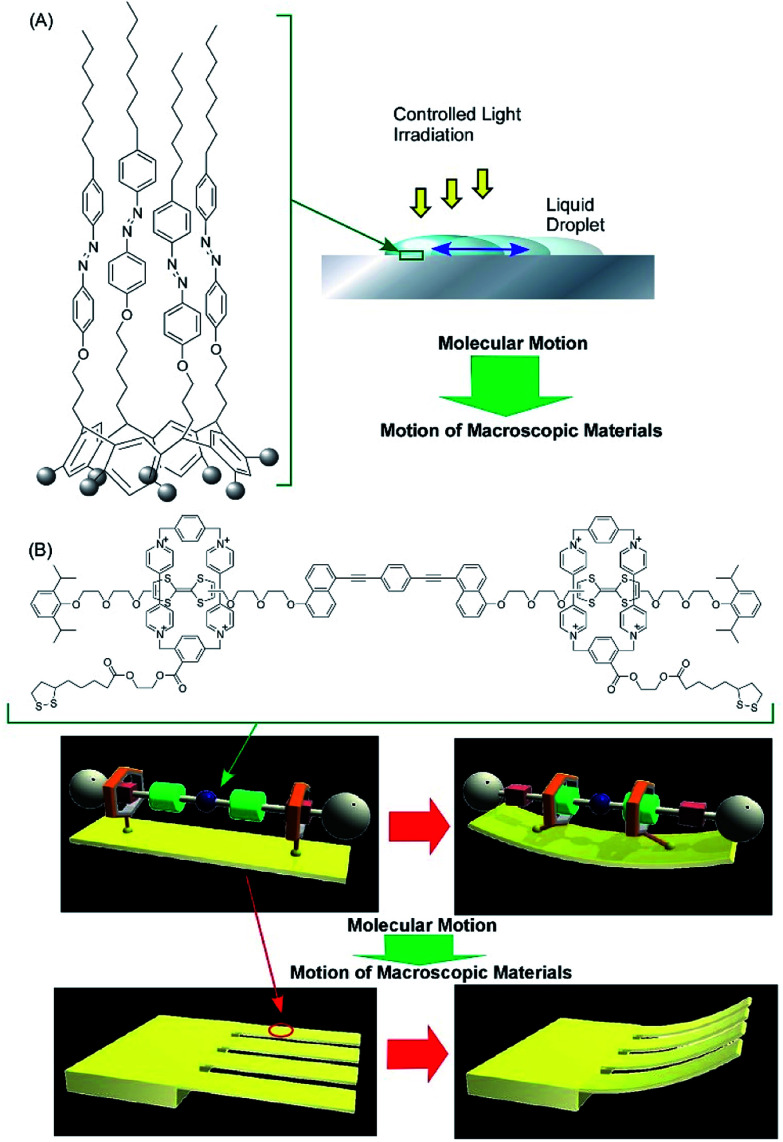
The accumulation and synchronization of motions of numerous molecular machines nanoarchitected on surface environments: (A) synchronized actions of the photo-isomerization of huge numbers of azobenzene arrays on a surface for controlling bulk liquids; and (B) the accumulation of molecular shuttle motions on a surface of cantilevers for the mechanical deformation of cantilevers.

**Fig. 7:**
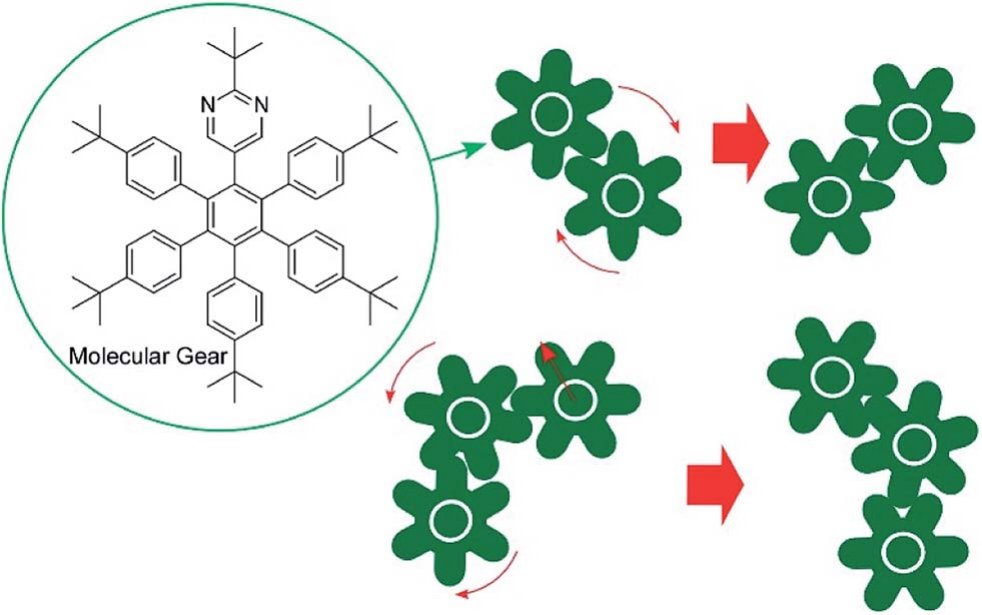
The train-like actions of single molecular gears on a Pb(111) surface.

The accumulation and synchronization of the motions of numerous molecular machines nanoarchitected on surfaces are rational ways to connect molecular-level motions to macroscopic mechanical material deformations. For example, the synchronized photo-isomerizations of huge numbers of azobenzene arrays on a surface can induce motion and orientation control of bulk liquids and liquid crystalline phases (Fig. 6A).^61^ The accumulation of molecular shuttle motions on a surface of cantilevers can induce mechanical deformation of much larger objects of the cantilevers (Fig. 6B).^62^ In more basic research, Soe *et al.* visually demonstrated train-like actions of single molecular gears on a Pb(111) surface using low-temperature STM (Fig. 7).^63^ The molecular gears with six teeth and a diameter of 1.2 nm are fixed on a single Cu adatom with an exact interval of 1.9 nm. Exact positioning between neighboring gears enables the transmission of gear rotations reversibly through tooth-to-tooth molecular mechanics. The concept of coupling gear motions could be extended to a molecular-level mechanical calculating machine.

## Molecular machines at dynamic liquid interfaces

**Fig. 8:**
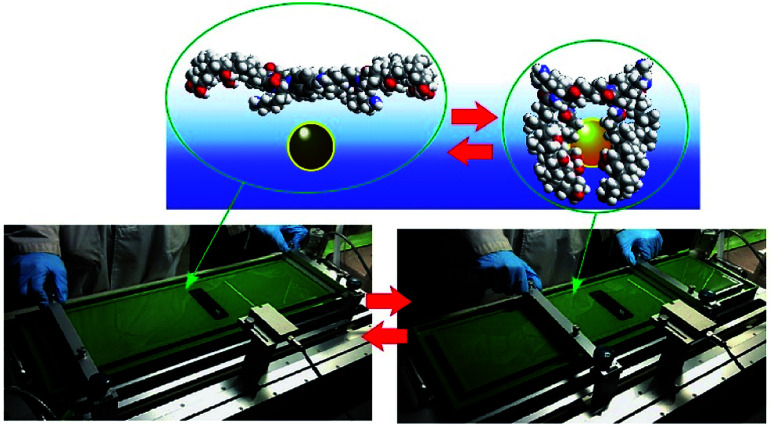
Cavity-flat conversions of a molecular catcher (steroid cyclophane) embedded at an air–water interface, accomplished through the compression and expansion of the monolayer.

Although clean solid surfaces are ideal environments to analyze detailed motions and functions of molecular machines, these media are not always adaptable for practical applications under ambient conditions. As more dynamically adaptable interfaces, liquid interfaces, such as the air–water interface, have been used for molecular machine functions. The air–water interface is actually an attractive medium for supramolecular chemistry, as it possesses several unique properties such as highly enhanced capabilities of molecular interaction and significantly shifted equilibrium.^64,65^ In addition, the extremely anisotropic nature of motional freedom at the air–water interface with macroscopically deformable lateral dimensions and a nanoscopically confined vertical dimension can couple macroscopic mechanical motions and molecular functions.^66–68^ The latter characteristics of the air–water interface enable us to operate molecular machine functions through hand-like macroscopic motions. For example, cavity-flat conversions of a molecular catcher (steroid cyclophane) embedded in an air–water interface can be repeatedly and reversibly accomplished through compression and expansion of their monolayer (Fig. 8).^69,70^ The capture and release of a target molecule by the molecular catcher can be controlled with hand-like macroscopic motions.

**Fig. 9:**
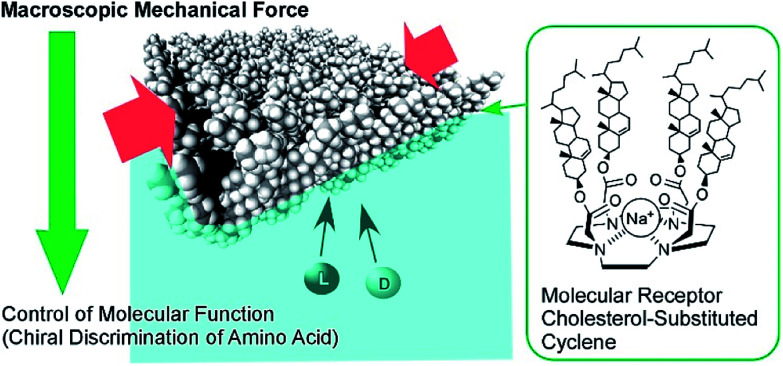
The *enantio*-selectivity control of aqueous amino acids through tuning the conformations of a molecular receptor, a cholesterol-armed cyclen complex, *via* mechanical compression.

**Fig. 10:**
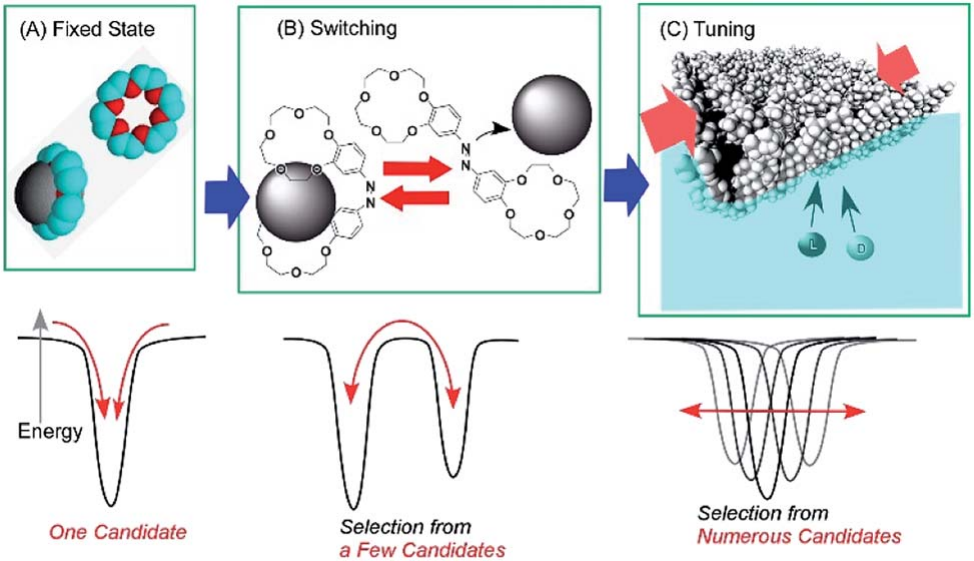
Molecular recognition modes: (A) fixed state mode; (B) switching mode; and (C) tuning mode.

The conformation of molecular receptors can be subtly tuned through mechanical processes to intentionally alter binding efficiency and selectivity to target guest molecules. For example, lateral compression of a monolayer of cholesterol-substituted triazacyclononane, as a molecular receptor for nucleic acid bases, optimizes discrimination between uracil and thymine derivatives,^71^ which cannot be distinguished by naturally occurring DNA and RNA. Reversion of *enantio*-selectivity of aqueous amino acids depending on lateral pressures was also realized through tuning the conformations of a molecular receptor, cholesterol-armed cyclen complex, by mechanical compression of the receptor monolayers (Fig. 9).^72^ In Fig. 10, the mechanical tuning of molecular receptors is compared with the traditional doctrines of molecular recognition. The basic principle of molecular recognition relies on the most stable complex structure between the host (receptor) and guest (Fig. 10A).^73–75^ If this can be regarded as the first generation of molecular recognition, the second generation of molecular recognition should be the switching of receptor structures by external stimuli, as seen in the pioneering example of photo-switchable azobenzene-type receptors by Shinkai (Fig. 10B).^76,77^ This switching mechanism utilizes two or more stable states to control recognition capability by external stimuli. Unlike these two traditional mechanisms, receptor tuning at a dynamic interface is based on selection and optimization from numerous conformational candidates of receptors (Fig. 10C).^78,79^ In this third-generation methodology, desirable efficiency and selectivity of molecular recognition can be tuned among numerous possibilities during continuous deformation of receptor structures.

**Fig. 11:**
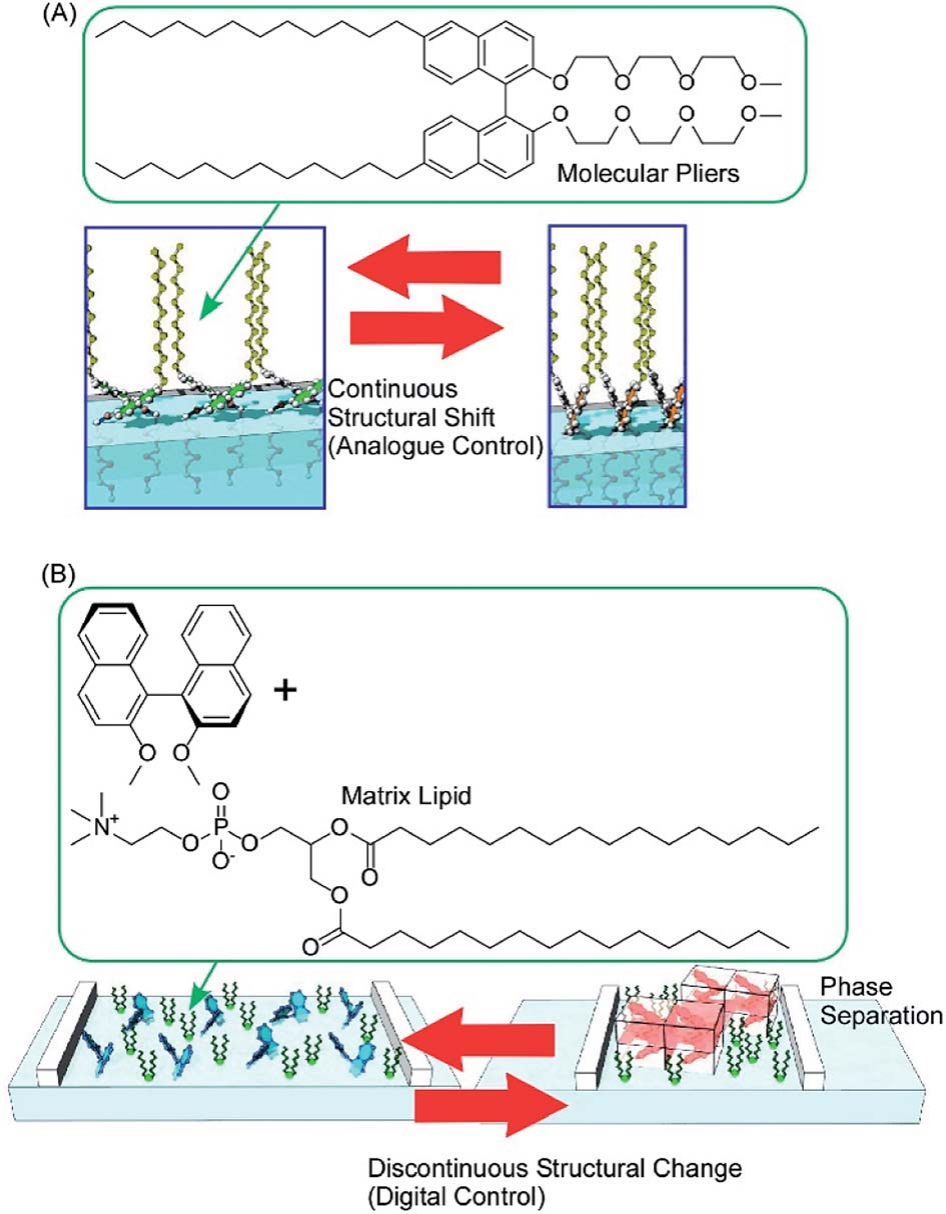
The conformation tuning of a binaphthyl unit: (A) analogue mode with molecular pliers; and (B) digital mode with a simple binaphthyl compound in a matrix lipid.

Conformation tuning of molecules can be done in both analogue mode and digital mode with interfacial nanoarchitectonics. The analogue tuning of the dihedral angles of the binaphthyl unit as a model unit was demonstrated by the continuous mechanical compression of a monolayer of molecular pliers, in which two oligo(ethylene oxide) hydrophilic heads and two alkyl hydrophobic tails are attached to the binaphthyl core (Fig. 11A).^80^ Mechanical compression of the molecular pliers monolayer induces continuous shifts of the dihedral angles of the binaphthyl unit. This analogue molecular tuning demonstrates that molecular conformations can be precisely manipulated using macroscopic lateral forces at the air–water interface. The applied forces are calculated to pN levels corresponding to those for delicate biological events such as DNA hybridization, myosin walking, and protein rotation and are much smaller than artificial processes such as photo-isomerization. When non-amphiphilic binaphthyl molecules are embedded within a monolayer of conventional amphiphilic molecules, the dihedral angles of the binaphthyl molecules are changed discontinuously upon compression of the mixed monolayer (Fig. 11B).^81^ The latter digital control of molecular conformation is accompanied by phase separation of binaphthyl crystals and dissolution of the crystals in a two-dimensional amphiphilic solvent. While analogue tuning of amphiphilic molecular pliers allows small continuous changes of the binaphthyl conformation in a cisoid to cisoid (closed to closed) transformation, the latter digital conversion is accompanied with the helical transformation of cisoid to transoid (closed to open, right to left) conversion.

**Fig. 12:**
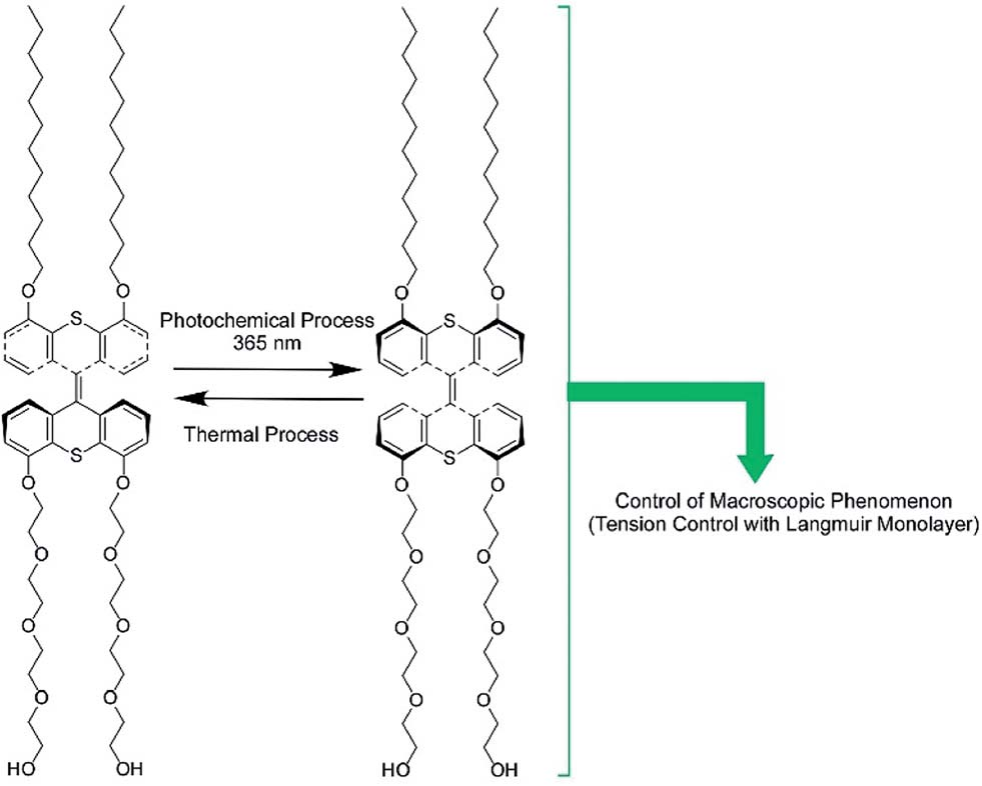
The bidirectional control of the surface tension of a macroscopic liquid surface through the photo-switching of a photoactive amphiphile with a bis(thiaxanthylidene) unit.

The regulation of molecular motions and conformations can be converted to macroscopic properties at a dynamic interface. Gengler, Feringa, and co-workers demonstrated bidirectional control of the surface tension of a macroscopic liquid surface (Fig. 12).^82^ They utilized photo-switching of a Langmuir monolayer of a photoactive amphiphile at the air–water interface. They used an amphiphile containing a bis(thiaxanthylidene) unit as a central core, with two hydrophilic tails and two hydrophobic tails at each side of the central core. Photoisomerization of the *anti*-folded to *syn*-folded geometry of the bis(thiaxanthylidene) unit as a central core induced up and down shifts of the surface tension of the water. Surface pressure increased upon photoisomerization at the two-dimensional low-density state, whereas a high-density state of the amphiphile resulted in a drop in surface pressure upon photo-irradiation, governed by the stacking state of the *syn*-folded conformers. Thus, the control of stacking states by addition of a matrix amphiphile, dipalmitoylphosphatidylcholine, modifies the regulation of surface tension. This means that not only is the photo-isomerization of responsive molecules an important factor for regulating surface tension, but the interfacial nanoarchitectonics of the components also are.

**Fig. 13:**
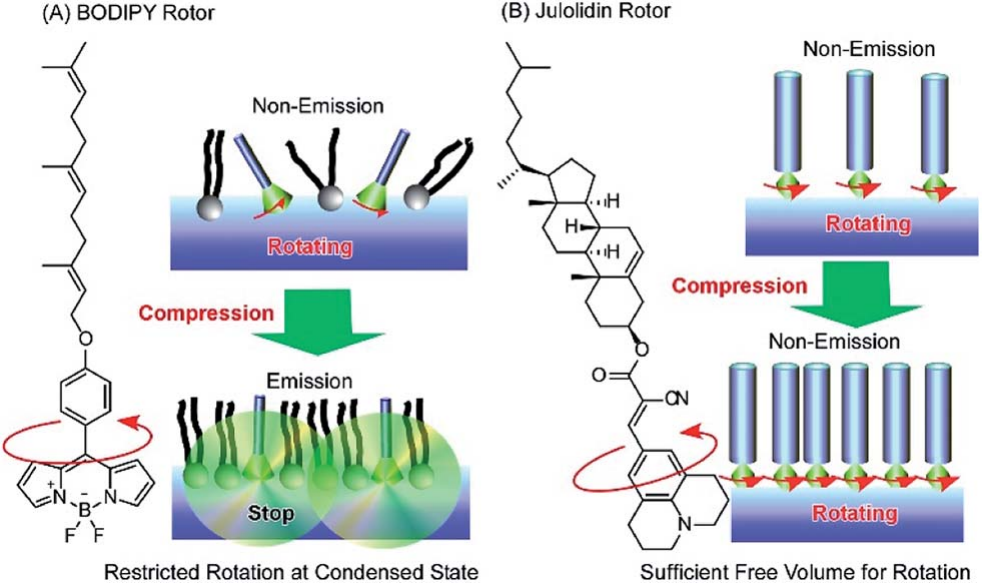
Molecular rotor behaviors at an air–water interface: (A) BODIPY rotor; and (B) julolidin rotor.

Photonic properties, such as light emission, are sometimes regulated by rotational motions of molecular rotors. Therefore, controlling the two-dimensional environments of molecular rotor motions using interfacial nanoarchitectonics can lead to regulated conversion of molecular motion into photonic outputs. At the air–water interface, the free volume for rotation of molecular rotors can be tuned by lateral pressures and co-existing matrix amphiphiles. In the case of the BODIPY-type molecular rotor, 4-farnesyloxyphenyl-4,4-difluoro-4-bora-3*a*,4*a*-diaza-*s*-indacene, the restriction of rotational motions results in fluorescence emission (Fig. 13A).^83^ With this molecular design, the cross-sectional area is not large enough to give sufficient free volume for BODIPY rotor rotation in the condensed state. Therefore, fluorescence emission from the BODIPY rotor can be controlled by various influential factors such as two-dimensional surface pressures and the compressibility of the matrix monolayer. In contrast, the rotation of another molecular rotor, a julolidine derivative with a cholesteryl hydrophobic moiety, is not hindered even in a highly condensed two-dimensional state at the air–water interface, because the bulky cholesteryl moiety reserves free volume for rotor rotation (Fig. 13B).^84^ However, the collapse of the two-dimensional ordered phase into a three-dimensional disordered phase results in the loss of rotational free volume, accompanied with fluorescence emission. These examples indicate that the nanoarchitectonics of ordered two-dimensional structures would provide many opportunities in the motional regulation of molecular machines.

**Fig. 14:**
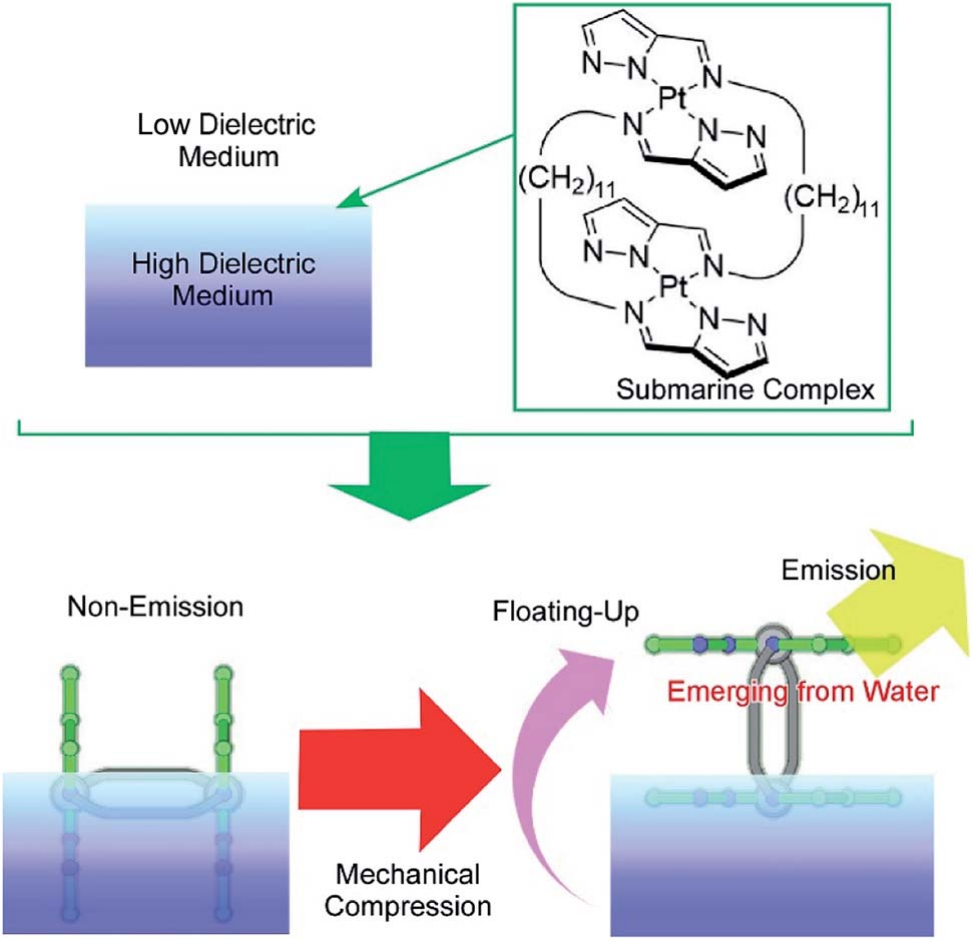
Submarine emission with a drastic increase of fluorescence emission through the floating-up motion of submarine molecules from the water phase into the air phase across a sub-nanometer level interfacial region.

Another specific feature of the air–water interface is a drastic change in dielectric nature across the interface. The control of molecular motion across the air–water interface can induce drastic changes in physical properties, with dependence on dielectric environments. One example was recently demonstrated as a novel concept called submarine emission (Fig. 14).^85^ In the submarine emission process, the floating-up motion of submarine molecules from the water phase into the air phase across a sub-nanometer level interfacial region induces a drastic increase of fluorescence emission. The submarine molecules employed are double paddle-type platinum dinuclear complexes, consisting of two mononuclear pyrazolato Pt(ii) complexes doubly linked by alkyl spacers. The H-shaped complexes of the submarine molecules are capable of taking various orientations at the air–water interface. At lower pressures, the submarine molecules are submerged in water, where they exhibit a weak emission. The submarine molecules are forced to float upward toward the air phase by mechanical compression of their monolayer. The weak interaction between the complex planes is broken so that one plane is exposed to the air phase. The complex plane in the air phase is free from dispersion of excitation energy due to molecular contacts under water and thus shows strong emission. Prior to mechanical compression of the monolayer of the submarine molecules, their phosphorescence is quenched but subsequently increases upon mechanical compression of the monolayer. Manipulation of the molecules at interfacial media with different dielectric constants would be an effective way to control their medium-induced photonic properties.

## Molecular machine functions in living cells and model membranes

Among various research efforts into molecular machines and related functional molecules, their usage in biological systems would be the most attractive. In particular, cell membranes provide many opportunities for molecular machines to express their potential capabilities.

### Membrane transport

One of the major roles of cell membranes is the regulation of chemical potential gradients between the inside and outside of the cell. The control of material transport by artificial molecules such as molecular carriers and channels has been researched with much interest.

**Fig. 15:**
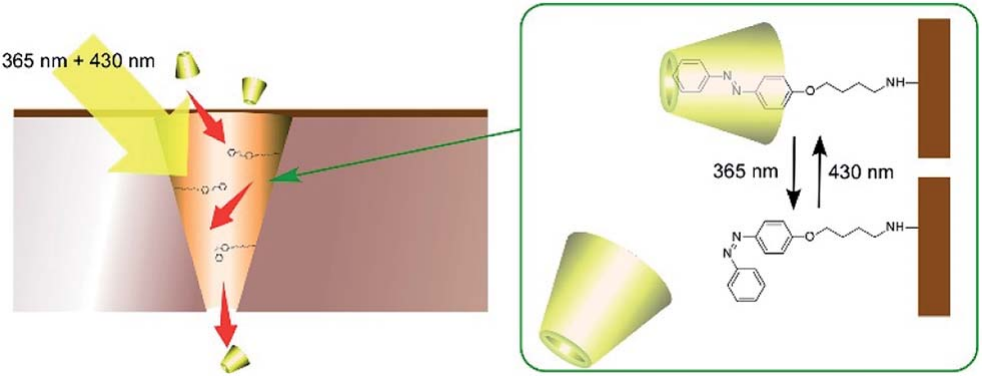
Photo-driven molecular motors for mass transport across model polymer membranes.

Molecular machines can be used for the regulation of material transport across the model membranes. Wen and co-workers demonstrated photo-driven molecular motors for mass transport across model polymer membranes (Fig. 15).^86^ On a polyimide membrane, conical-shape nanochannels were first fabricated with ion-track etching. The inside surfaces of the nanochannels were then modified with photo-sensitive azobenzene-type molecular motors. The modified nanochannels were found to be capable of autonomous transport of selective guest molecules through simultaneous irradiation with UV and visible light. In the case of β-cyclodextrin transport, β-cyclodextrin is trapped by the *trans*-form of azobenzene upon visible-light irradiation and the trapped β-cyclodextrin is released by UV light irradiation. Interestingly, balanced intensity between UV and visible light can facilitate mass transport better than strong intensity of a single wavelength.

**Fig. 16:**
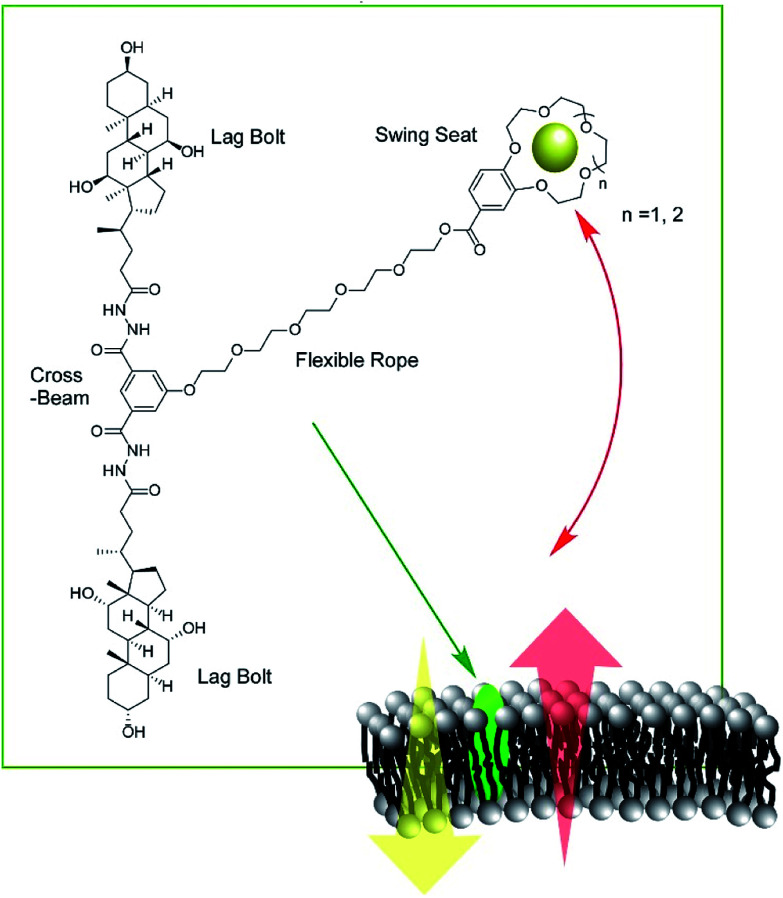
Molecular swings, for ion transport across model lipid bilayer membranes.

Zeng and co-workers developed a new type of molecular machine, a molecular swing, for ion transport across model lipid bilayer membranes (Fig. 16).^87^ The molecular swing has a 15-crown-5 part as a carrier unit with ion binding and transport capabilities, together with a lag bolt component to anchor the swing into the hydrophilic regions of the membrane. Even in the cholesterol-rich conditions of lipid membranes, high ion transport behavior was observed. The ion transport mechanism is based on numerous pathways, making the flux for transmembrane K^+^ transport highly efficient, corresponding to 27% better than that for gramicidin A. Anticancer activity can even be achieved by further structure optimization.

As reported by Zhang, Long, and co-workers, the motion of a DNA molecular train with azobenzene gears within aerolysin pores was evaluated with high temporal and spatial resolution through electrical signals.^88^ A cytolytic pore-forming toxin known as aerolysin is embedded in lipid membranes. Photo-isomerization of azobenzene attached to the molecular train can modulate the speed of the train in the aerolysin pore. Based on such motional control of molecular machines in biological pores, single-molecule computing may become possible upon logic operation with selected inputs of multiple external stimuli.

### Drilling cell membrane

**Fig. 17:**
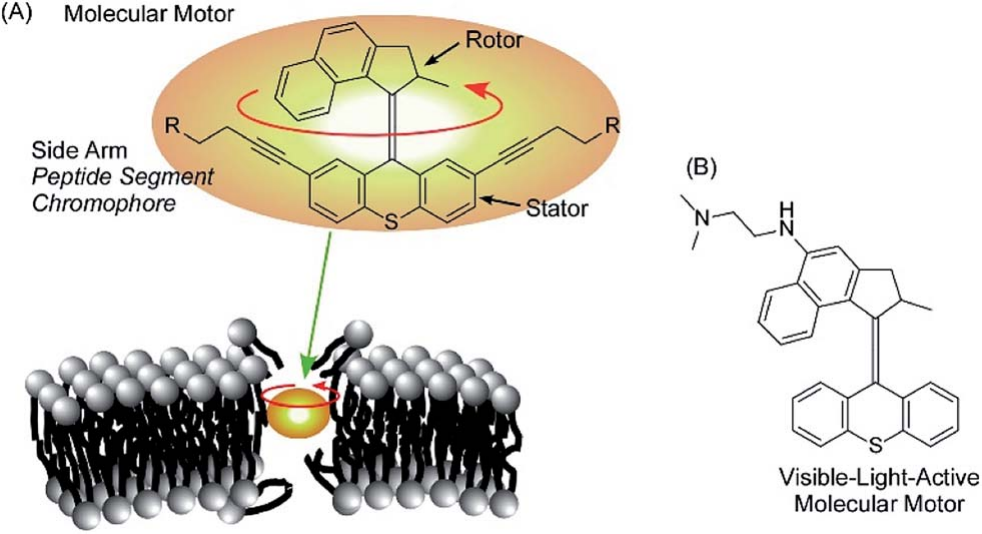
The hole opening of cell membranes through the drilling effect of molecular motors (A) and a visible-light-active version of a motor (B).

Rotational motion of well-designed molecular motors can induce hole opening in cell membranes through a drilling effect, as demonstrated by García-López *et al.* (Fig. 17A).^89^ Molecular motors are adsorbed on the membranes of living cells, and conformational changes of the molecular motors upon application of external stimuli such as UV light irradiation induce disturbances that open holes in the cell membranes. Diffusion of only a trace amount of molecular motors can lead to necrosis and diffusion of outer chemicals into live cells. By introducing short peptide segments to the arms of the molecular motor, the mechanical actions of the molecular motor selectively occurred at target cells with corresponding recognition sites. Because the size of the molecular motors is at the 1 nm level, ruptures of thicker cell membranes by nanomechanical effects are not always immediate. Concerted motions of 1 nm size motors can dislocate membrane components significantly.

Orozco *et al.* developed more useful systems. Their molecular motor can be activated with much safer visible light instead of UV light (Fig. 17B).^90^ Pancreatic cancer cells are actually killed by the newly synthesized molecular motors with visible light irradiation (405 nm light). The proposed system could be applied to skin cancer cells because the affected areas can be easily exposed to visible light. Unlike healthy cells, cancer cells lack a protective stratum corneum layer. Therefore, artificial molecular motors are expected to adsorb selectively on cancer cells.

### Coupling with bio-machines

**Fig. 18:**
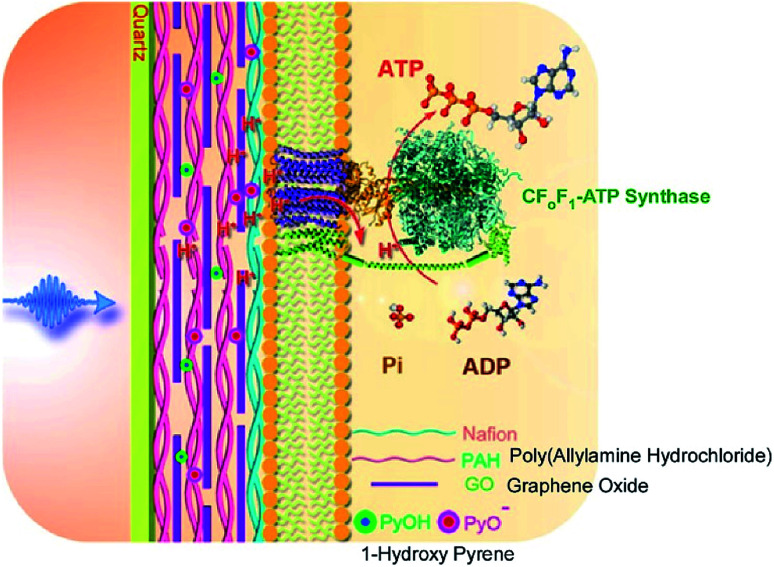
Layer-by-layer structures with a motor protein together with a photoacid generator and proton conductor for photo-driven ATP synthesis. Reproduced with permission ref. 91. Copyright 2017, American Chemical Society.

In addition to introducing artificial molecular machines into biological systems, the introduction of biomolecular machines into artificial supramolecular systems has become a hot topic. In natural biological systems, there are many kinds of highly complex biomolecular machines operating at biological interfacial media, such as cell membranes. Therefore, nanoarchitecting biomolecular machines into supramolecular films is a rational approach to generating bio-mimetic functional systems. For example, Li and co-workers successfully immobilized a motor protein together with a photoacid generator and proton conductor in a layer-by-layer structural motif (Fig. 18).^91^ On a quartz slide, a multilayer layer-by-layer film of poly(allylamine hydrochloride) and graphene oxide is first assembled, into which 1-hydroxy pyrene molecules as photoacid generators are loaded through π–π interactions with graphene oxide. Nafion is next coated as a proton conductor on the surface of the layer-by-layer film, and then proteoliposomes containing the motor protein, ATP synthase, are spread on top. The nanoarchitected artificial membrane with biomolecular motors can work as photo-activated ATP producer. Photo-irradiation deprotonates 1-hydroxy pyrene to release protons that can transmit though the membrane *via* the proton conductor. The generated proton gradient activates the motor proteins to synthesize ATP molecules.

**Fig. 19:**
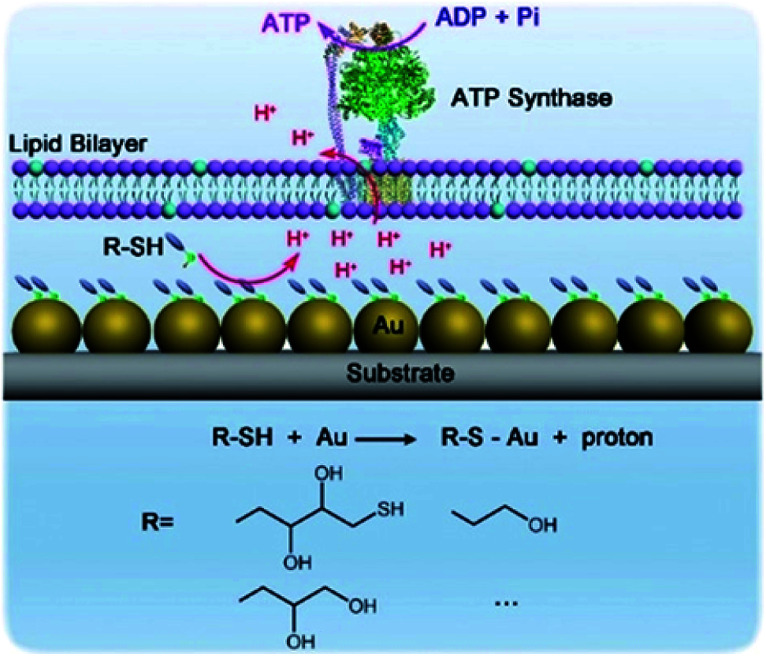
Hybrid systems using gold nanoparticles and motor protein ATP synthase to expand bioenergy processes to include self-assembled monolayer chemistry. Reproduced with permission ref. 92. Copyright 2019, Wiley-VCH.

Hybrid nanoarchitectonics with artificial nanostructures and biomolecular motors can expand categories of molecular fuels. Li and co-workers constructed hybrid systems using gold nanoparticles and the motor protein ATP synthase to expand bioenergy processes to include self-assembled monolayer chemistry (Fig. 19).^92^ Gold nanoparticles were immobilized on a glass substrate through magnetron sputtering, onto which proteoliposomes containing ATP synthase were spread to complete the hybrid system. Binding added alkane thiol molecules on a surface of gold nanoparticles to form a self-assembled monolayer induced the release of protons. This resulted in a proton gradient across the adjacent membrane, which formed the driving energy to rotate ATP synthase and activate ATP production. The bio-irrelevant supramolecular process, self-assembled monolayer formation, can behave like a bio-energy production process. Well-considered nanoarchitectonics efforts to couple biological molecular machines with artificial nanostructured systems could create possibilities to include various chemical processes into biomimetic energy and material conversion systems.

## Short perspective for a long future

Molecular machines were initially studied as toys in the molecular sciences. Their on-demand motions are evaluated as averaged behaviors in their solutions. Advanced nanotechnology apparatus such as STM allows us to observe and manipulate molecular machines directly. Molecular machines can be visualized as photos and movies. However, these fundamental studies may not be enough to convert molecular machines from scientific toys to useful tools.

One important key for the practical usage of molecular machines is organizing them in well-defined media. Highly sophisticated research on solid surfaces has revealed that molecular rotors are capable of synchronized and directional motions upon their association.^93,94^ It was also demonstrated that dynamic media such as Langmuir monolayers and their transferred films can host functions of rotaxanes.^95^ Appropriate working media for molecular machines must be able to accept external stimuli and emit output dynamically. Dynamic interfaces would be appropriate media for this second stage of molecular machine research. Upon embedding molecular machines at the dynamic air–water interface, molecular machine functions such as molecular capture, rotation, and submarine emission are connected with macroscopic mechanical motions that are regarded as practical inputs. As more advanced examples, molecular machine operations have been recently demonstrated at biomolecular interfaces, including living cell surfaces and lipid bilayer membrane models. Science-fiction-like dreams of ultra-small machines have been realized, as seen in the recent example where artificial molecular motors drill open pores in cancer cell membranes. Molecular machines have surely become useful tools.

Machine-like mechanisms are ubiquitous within life forms and carry out sophisticated functions. Indeed, organisms may be regarded as a huge organization of biomolecular machines. Life forms with biomolecular machines are the products of billions of years of evolutionary processes. We may nanoarchitect such highly complex and sophisticated functional systems with artificial molecular machines within much shorter periods. Further developments of molecular machines as useful tools require some additional research to increase their capabilities. Achieving motional and functional relays of multiple molecular machines nanoarchitected at interfaces would be an important step to realize the sophisticated conversion of materials, energy, and information. Cooperative work to apply functional molecules to cell membranes towards achieving molecular factories is a common strategy in biological research. These high functions could be mimicked through the organization of artificial molecular machines at interfacial environments. Another important factor for realizing the practical usage of molecular machines is achieving a connection between molecular-level machine outputs and practical-level macroscopic signals. Signal amplification through the concerted actions of molecular machines is a possible solution. The rational accumulation of concerted actions of molecular machines can be done through organizing assemblies of molecular machines at interfacial media. The evolution of molecular machines from toys to tools would be supported by interfacial nanoarchitectonics approaches.

## Conflicts of interest

There are no conflicts to declare.
